# Ginsenoside Rb1 Mitigates *Escherichia coli* Lipopolysaccharide-Induced Endometritis through TLR4-Mediated NF-κB Pathway

**DOI:** 10.3390/molecules26237089

**Published:** 2021-11-23

**Authors:** Aftab Shaukat, Irfan Shaukat, Shahid Ali Rajput, Rizwan Shukat, Sana Hanif, Imran Shaukat, Xinxin Zhang, Chao Chen, Xuyang Sun, Tingzhu Ye, Kaifeng Niu, Zhiqiu Yao, Shadab Shaukat, Muhammad Safdar, Mohamed Abdelrahman, Umair Riaz, Junwei Zhao, Xiaoying Gu, Liguo Yang

**Affiliations:** 1 National Center for International Research on Animal Genetics, Breeding and Reproduction (NCIRAGBR), Huazhong Agricultural University, Wuhan 430070, China; aftabshaukat40@gmail.com (A.S.); zxx1144795936@163.com (X.Z.); chenchao1995@webmail.hzau.edu.cn (C.C.); sunxuyangabc@163.com (X.S.); yetingzhu@webmail.hzau.edu.cn (T.Y.); nkf_19930806@163.com (K.N.); zhiqiuyao1@163.com (Z.Y.); safdar1126@webmail.hzau.edu.cn (M.S.); Mohamed.Asad@agr.au.edu.eg (M.A.); umair.riaz@iub.edu.pk (U.R.); zjwzjw98@163.com (J.Z.); gxy19970310@163.com (X.G.); 2 Faculty of Medicine, University of Lorraine, 54052 Nancy, France; irfan-uaf@hotmail.com; 3 Faculty of Veterinary and Animal Sciences, Muhammad Nawaz Shareef University of Agriculture, Multan 66000, Pakistan; shahid.ali@mnsuam.edu.pk or; 4 Faculty of Food, Nutrition & Home Sciences, University of Agriculture, Faisalabad 38000, Pakistan; rizwanuaf@hotmail.com; 5 Hubei Key Laboratory of Theory and Application of Advanced Materials Mechanics, Wuhan University of Technology, Wuhan 430070, China; Sanahanif96@gmail.com; 6 Department of Physics, University of Agriculture, Faisalabad 38000, Pakistan; imran.uaf1349@gmail.com; 7 Department of Plant Breeding and Genetics, University of Agriculture, Faisalabad 38000, Pakistan; shadab_uaf@hotmail.com; 8 Animal Production Department, Faculty of Agriculture, Assuit University, Asyut 71515, Egypt

**Keywords:** endometritis, lipopolysaccharide, Ginsenoside Rb1, TLR4, NF-κB pathway

## Abstract

Endometritis is the inflammatory response of the endometrial lining of the uterus and is associated with low conception rates, early embryonic mortality, and prolonged inter-calving intervals, and thus poses huge economic losses to the dairy industry worldwide. Ginsenoside Rb1 (GnRb1) is a natural compound obtained from the roots of *Panax ginseng*, having several pharmacological and biological properties. However, the anti-inflammatory properties of GnRb1 in lipopolysaccharide (LPS)-challenged endometritis through the TLR4-mediated NF-κB signaling pathway has not yet been researched. This study was planned to evaluate the mechanisms of how GnRb1 rescues LPS-induced endometritis. In the present research, histopathological findings revealed that GnRb1 ameliorated LPS-triggered uterine injury. The ELISA and RT-qPCR assay findings indicated that GnRb1 suppressed the expression level of pro-inflammatory molecules (TNF-α, IL-1β and IL-6) and boosted the level of anti-inflammatory (IL-10) cytokine. Furthermore, the molecular study suggested that GnRb1 attenuated TLR4-mediated NF-κB signaling. The results demonstrated the therapeutic efficacy of GnRb1 in the mouse model of LPS-triggered endometritis via the inhibition of the TLR4-associated NF-κB pathway. Taken together, this study provides a baseline for the protective effect of GnRb1 to treat endometritis in both humans and animals.

## 1. Introduction

Endometritis is considered a major global problem and has been associated with a decline in the reproductive performance of animals [1,2]. The postpartum involution period is very crucial regarding the infection of the endometrium caused by bacteria, especially Gram-negative (G-ve) bacteria [3]. Among these G-ve bacteria, *Escherichia coli* (*E. coli*) is known as the major bacterial etiology of endometritis [4,5]. The endometrial lining of the uterus is the first line of defense that recognizes the pathogen-associated molecular pattern (PAMP) from invading pathogens to activate immunity [6]. Toll-like receptor (TLR) 4 is a major PAMP activated by lipopolysaccharide (LPS) [7]. LPS is a potent immune stimulator derived from the outer membrane of G-ve bacteria [8]. Once TLR4 is activated, it triggers the upregulation of underlying inflammatory pathways, especially the NF-κB signaling pathway [5]. Activated TLR4-mediated NF-κB results in the production of pro-inflammatory cytokines that play a role in inflammation [9,10].

**Figure 1:**
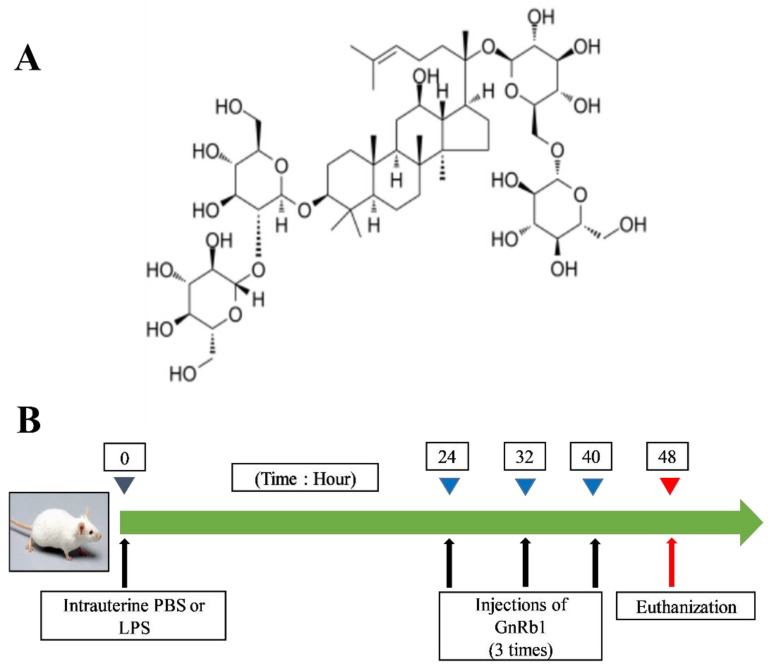
(**A**) The structure of Ginsenoside Rb1. (**B**) Animal treatment protocol of this study.

Antibiotics are used to treat endometritis globally. However, bacterial resistance and food safety issues are the major concerns to treating endometritis with antibiotics [4]. Therefore, alternative therapeutic agents are in dire need in the current era. Ginsenoside Rb1 (GnRb1) has well-known anti-inflammatory properties (Figure 1A). GnRb1 was found to suppress *Staphylococcus* (*S*.) *aureus*-triggered inflammatory responses in vivo and in vitro via the TLR2-mediated NF-κB pathway [11]. In addition, GnRb1 mitigated LPS-caused acute lung injury (ALI) in rats [12]. Intraperitoneal Rb1 therapy suppresses the levels of the pro-inflammatory markers and attenuates NF-κB molecules (p-IκBα and p-IKK) in vivo model [13]. In addition, GnRb1 treatment significantly ameliorated LPS-induced microglial inflammation and suppressed the production of pro-inflammatory cytokines [14]. Zhu et al. [15] explored the neuroprotective effects of GnRb1 in a rat model of ischemic neuro-inflammation. However, whether GnRb1 has protective effects in LPS-induced endometritis still remains unexplored. Therefore, the present research was planned to explore the beneficial effects of GnRb1 in the murine model of endometritis caused by LPS.

## 2. Materials and Methods

### 2.1. Ethical Approval

Experiments regarding animals were carried out after being approved by the institutional research ethics and animal welfare committee, Huazhong Agricultural University (HZAUMO_2015-12) (Wuhan, China).

### 2.2. Chemicals and Reagents

GnRb1 was attained from Shanghai Yuanye Bio-Technology Co., Ltd. (Shanghai, China). LPS from *E. coli* strain (O55:B5) was obtained from Sigma (St. Louis, MO, USA). The ELISA kits to determine MPO, pro- and anti-inflammatory cytokines were obtained from Nanjing Jiancheng Bioengineering Institute (Nanjing, China). ELISA kits NF-κB pathway (total and phospho IκBα and NF-κB p65) proteins and primary, and secondary antibodies were procured from Cell Signaling Technology (CST, Danvers, MA, USA).

### 2.3. Animal and Experimental Groups

Sixty female BALB/c mice of 25 ± 2 g weight were procured from Wuhan University (Wuhan, China). The mice were arbitrarily divided into four (*n* = 15) groups as follows:

1. Control group (50 µL of Saline solution);
2. LPS group (a volume of 50 µL having a concentration of 1 mg/mL);
3. LPS + GnRb1 (25 mg/kg) group;
4. LPS + GnRb1 (50 mg/kg) group.

LPS-induced endometritis was established by intrauterine infusion of 50 µL of LPS (1 mg/mL) according to the previous study [4,7]. After 24 h, mice were injected intraperitoneally with GnRb1 (25 and 50 mg/kg) thrice, 8 h apart. Eight hours after the last GnRb1 injection, mice were euthanized to collect uterine samples. The animal treatment protocol of this study is displayed in (Figure 1B).

### 2.4. Histological Assay

The uterine tissue sections were placed in a 10% formalin solution for 2 days for tissue fixation purposes. These sections were then dehydrated and embedded in liquid paraffin. The samples were dewaxified and cut into 4 to 5 µm slices by microtome. Subsequently, uterine slices were stained with hematoxylin and eosin (H&E).

### 2.5. Wet to Dry (W/D) Weight Ratio MPO Assay

Uteri were washed thrice with PBS and weighed; the weight was recorded as wet weight. Formerly, uteri were retained in an oven of 80 °C for 24 h; the weight was recorded as dry weight. The uterine edema was calculated as W/D ratio. The uterine tissues were homogenized (*w*/*v*, 1/9) to measure the MPO activity assay according to supplier instructions.

### 2.6. ELISA Assay

The levels of pro-inflammatory markers (IL-1β, IL-6 and TNF-α), anti-inflammatory (IL-10) cytokines, and NF-κB pathway (total and phospho IκB- α and NF-κB p65) in uterine tissue were measured in triplicate by ELISA kits following manufacturer’s guidelines.

### 2.7. Real-Time Quantitative RCR Assay

Total RNA from uterine tissue was harvested by TRIzol (Invitrogen, Carlsbad, CA, USA). The complementary DNA was then synthesized from extracted RNA. The expression of TLR4, pro, and anti-inflammatory mediators were detected and quantified by using SYBR Green Master Mix (TaKaRa Biotechnology, Tokyo, Japan) according to guidelines provided by the manufacturer. The primers employed in this study are mentioned in Table 1. GAPDH was used as a control. The data values were calculated using the 2^−^^ΔΔ^^CT^ method according to the previous study [16].

### 2.8. Western Blot

The total protein from uterine tissue was harvested using a RIPA lysis solution (Beyotime Biotechnology, Shanghai, China) containing a phosphatase inhibitor. A BCA kit (Vazyme Biotech, Nanjing, China) was used for the determination of protein concentration. Subsequently, equal quantities in terms of protein concentration were loaded and fractionated on SDS-PAGE. Next, the protein was transfer to PVDF membrane. Nonprotein parts of the membranes were blocked by skimmed milk (5%) for 2 h. Then, the membranes were probed with primary antibodies at 4 °C overnight. The TBST was washing of membranes (thrice; 10 min each). The membranes were incubated with a secondary antibody for 1 h at room temperature. The expression profile of protein was measured by an ECL Plus Western blot detection system.

### 2.9. Statistical Analysis

Three independent replicates were used in each experiment. GraphPad Prism 8.02 (San Diego, CA, USA) was used for the assessment of data. One-way analysis of variance (ANOVA) followed by Dunnet’s multiple comparison test were employed in a Gaussian distribution pattern. The data values are mentioned as mean ± Standard Error of Mean (S.E.M.). The *p*-value of (<0.05) was considered statistically significant.

## 3. Results

### 3.1. GnRb1 Alleviates LPS-Induced Murine Endometritis

**Figure 2:**
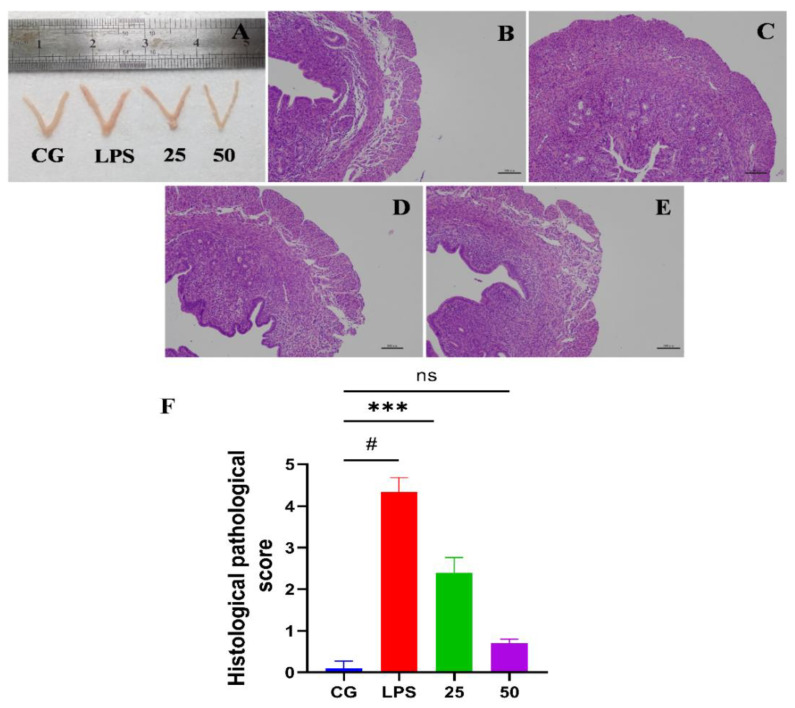
Effects of GnRb1 against LPS-triggered uterine injury. (**A**) The morphology of uterine tissue. (**B**) The control group (CG). (**C**) The LPS group. (**D**,**E**) The LPS + GnRb1 (25 and 50 mg/kg, respectively). (**F**) Histopathological score of uterine sections. The scale bar is of 100 µm (200× magnification). CG represents the control group. LPS reveals the LPS-induced groups. The 25 and 50 are the GnRb1-administrated groups representing 25 mg/kg and 50 mg/kg per animal, respectively. GnRb1 indicates the Ginsenoside Rb1. Data statistics are demonstrated as mean ± S.E.M. The # *p* < 0.001 CG versus LPS group, *** *p* < 0.001 and ns indicates the nonsignificant difference between CG versus LPS + GnRb1 groups.

The effect of GnRb1 against LPS-triggered uterine injury can be obviously seen in Figure 2A. No histopathological alterations were observed in the control group (Figure 2B). However, pathological changes, i.e., hyperemia, hemorrhages, edema, and penetration of inflammatory cells, were detected in the LPS-administrated group (Figure 2C). The LPS-caused histopathological alterations were drastically alleviated by GnRb1 at the dose rate of 25 and 50 mg/kg (Figure 2D,E). To confirm these findings, a histopathological score was recorded according to the previous study [17] (Figure 2F). Accumulatively, these findings show that GnRb1 efficiently ameliorated LPS-stimulated endometritis.

### 3.2. Effect of GnRb1 against LPS-Induced MPO Activity and W/D Ratio

**Figure 3:**
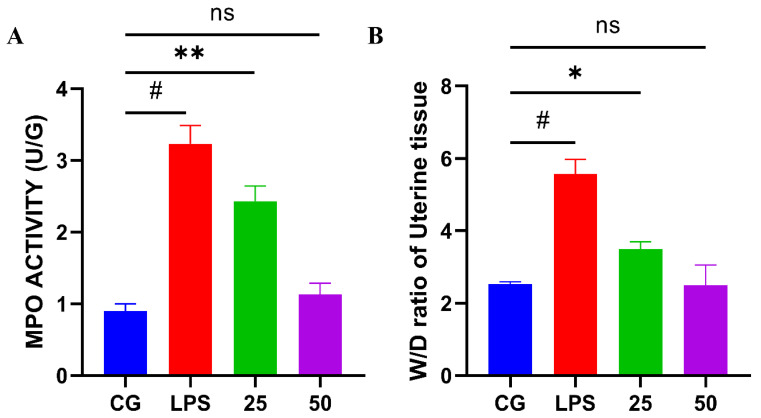
Consequence of GnRb1 against MPO activity assay and W/D ratio in mice uterus. (**A**) MPO (U/G) activity. (**B**) W/D ratio. CG represents the control group. LPS reveals the LPS-induced groups. The 25 and 50 are the GnRb1-administrated groups representing 25 mg/kg and 50 mg/kg per animal, respectively. GnRb1 indicates the Ginsenoside Rb1. MPO indicates the myeloperoxidase, whereas W/D depicts the wet to dry weight ratio. Data statistics are demonstrated as mean ± S.E.M. The # *p* < 0.001 CG versus LPS group, * *p* < 0.05, ** *p* < 0.01, and ns indicates the nonsignificant difference between CG versus LPS + GnRb1 groups.

MPO activity is a marker of neutrophilic (the first line of defense) penetration. The results revealed that LPS immensely (*p* < 0.05) boosted the MPO activity in uterine tissue compared to the control group. Nevertheless, GnRb1 therapy sharply (*p* < 0.05) decreased the MPO activity, as indicated in Figure 3A. Edema is a peculiar sign of LPS-mediated inflammation [10]. The W/D ratio of uterine tissue was measured to uterine edema. The W/D ratio was significantly (*p* < 0.05) improved in the LPS group compared with the control group. However, the uterine W/D ratio was reduced significantly (*p* < 0.05) in both GnRb1-treated (25 and 50 mg/kg) groups, as shown in Figure 3B.

### 3.3. Effect of GnRb1 against LPS-Induced Expression of Pro- and Anti-Inflammatory Markers

**Figure 4:**
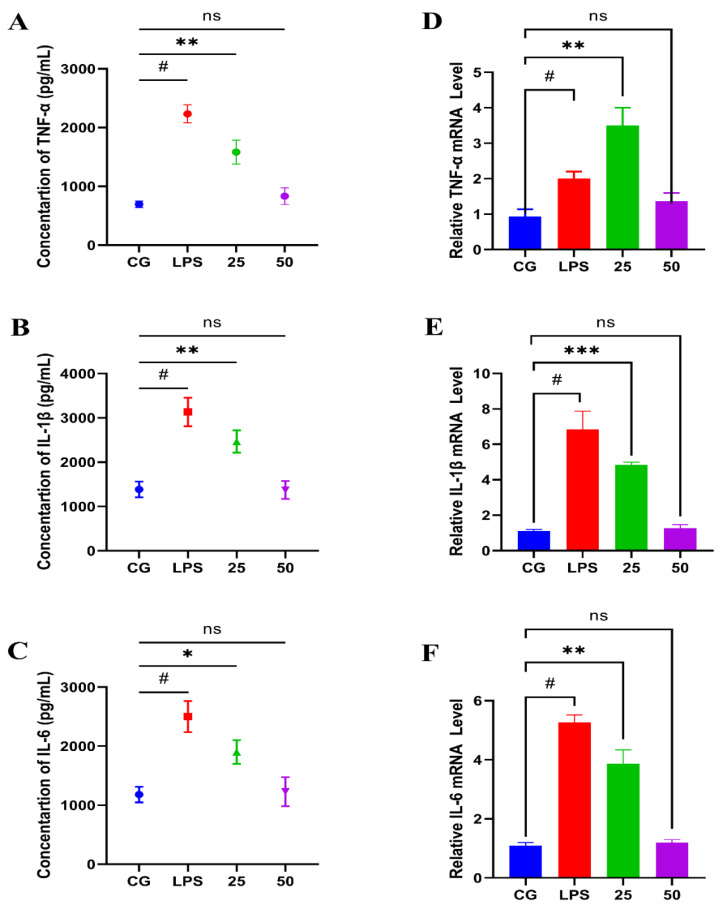
Effects of GnRb1 against LPS-induced production of pro-inflammatory cytokines. The concentration of: (**A**) the TNF-α (pg/mL); (**B**) the IL-1β (pg/mL); and (**C**) IL-6 (pg/mL). The relative mRNA expression levels of (**D**) TNF-α, (**E**) IL-1β; and (**F**) IL-6. CG represents the control group. LPS reveals the LPS-induced groups. The 25 and 50 are the GnRb1-administrated groups representing 25 mg/kg and 50 mg/kg per animal, respectively. GnRb1 indicates the Ginsenoside Rb1. Data statistics are demonstrated as mean ± S.E.M. The # *p* < 0.001 CG versus LPS group, * *p* < 0.05, ** *p* < 0.01, *** *p* < 0.001, and ns indicates the nonsignificant difference between CG versus LPS + GnRb1 groups.

Pro- and anti-inflammatory mediators are directly involved in the development of endometritis [18]. To investigate the protective properties of GnRb1, the concentration and gene expression level of pro-inflammatory mediators have been measured by ELISA and RT-qPCR assay, respectively. The findings of the ELISA assay indicated that GnRb1 therapy significantly (*p* < 0.05) suppressed the concentrations of TNF-α (Figure 4A), IL-1β (Figure 4B), and IL-6 (Figure 4C), which were increased by LPS. These findings were further confirmed by gene expression via an RT-qPCR assay. Interestingly, a similar trend was noticed as a result of the ELISA assay. Compared with the control group, the LPS group obviously (*p* < 0.05) increased the gene expressions of TNF-α (Figure 4D), IL-1β (Figure 4E), and IL-6 (Figure 4F), which were attenuated significantly (*p* < 0.05) by GnRb1 treatment in a dose-dependent manner.

**Figure 5:**
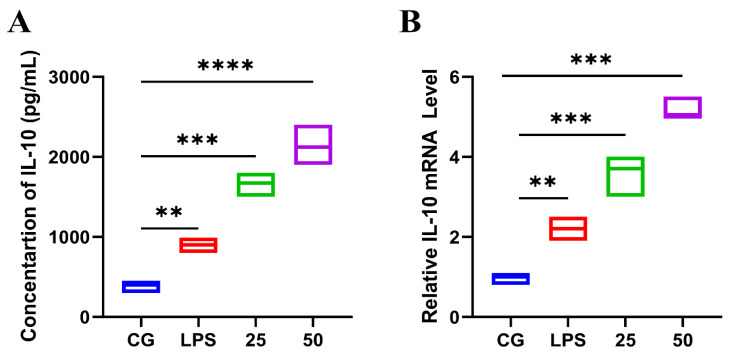
Effects of GnRb1 against LPS-induced production IL-10 concentration. The concentration of (**A**) IL-10 (pg/mL). The relative mRNA expression level of (**B**) IL-10 in uterine tissue. CG represents the control group. LPS reveals the LPS-induced groups. The 25 and 50 are the GnRb1-administrated groups representing 25 mg/kg and 50 mg/kg per animal, respectively. GnRb1 indicates the Ginsenoside Rb1. Data statistics are demonstrated as mean ± S.E.M. The ** *p* < 0.001 CG versus LPS group, ** *p* < 0.01, *** *p* < 0.001, **** *p* < 0.0001, and ns indicates the nonsignificant difference between CG versus LPS + GnRb1 groups.

Conversely, GnRb1 increased (*p* < 0.05) the concentration of the IL-10 in LPS-administrated groups (Figure 5A), as well as the mRNA expression level of IL-10 (Figure 5B) in a dose-dependent pattern. These findings indicated that GnRb1 attenuated the production of pro-inflammatory mediators and boosted anti-inflammatory cytokine production.

### 3.4. GnRb1 Represses LPS-Induced TLR4 Expression

**Figure 6:**
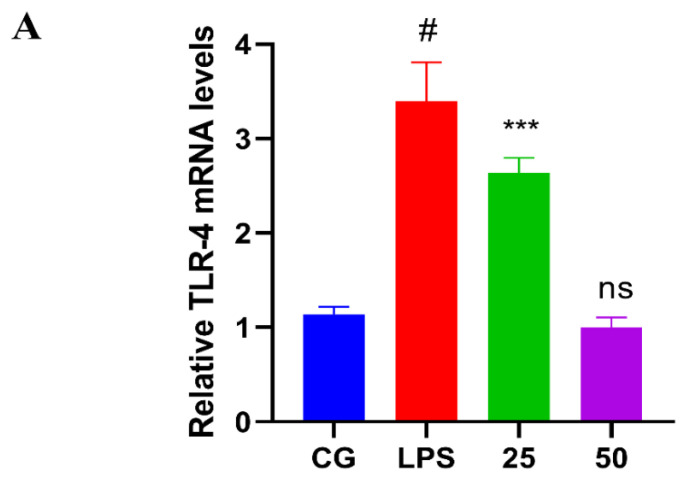

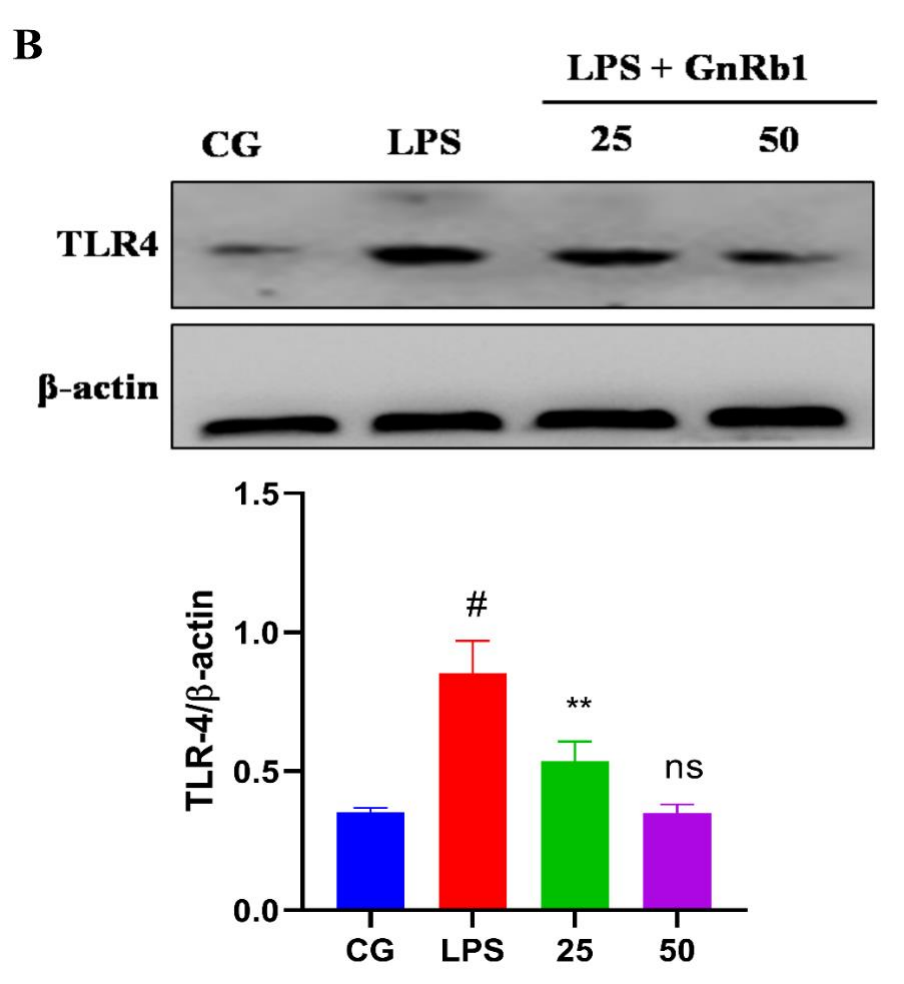
Effects of GnRb1 against LPS-induced expression of the TLR4. (**A**) The relative mRNA expression level of the TLR4 gene. (**B**) The protein expression of TLR4. CG represents the control group. LPS reveals the LPS-induced groups. The 25 and 50 are the GnRb1-administrated groups representing 25 mg/kg and 50 mg/kg per animal, respectively. GnRb1 indicates the Ginsenoside Rb1. Data statistics are demonstrated as mean ± S.E.M. The # *p* < 0.001 CG versus LPS group, ** *p* < 0.01, *** *p* < 0.001, and ns indicates the nonsignificant difference between CG versus LPS + GnRb1 groups.

TLR4 is activated as the host defense mechanism against LPS. As displayed in the results of RT-qPCR (Figure 6A) as well as Western blot assay (Figure 6B), TLR4 expression was dramatically increased (*p* < 0.05) in the LPS group. However, GnRb1 therapy suppressed (*p* < 0.05) the LPS-stimulated TLR4 expression.

### 3.5. GnRb1 Suppresses LPS-Induced Activation of NF-κB Signaling Pathway

**Figure 7:**
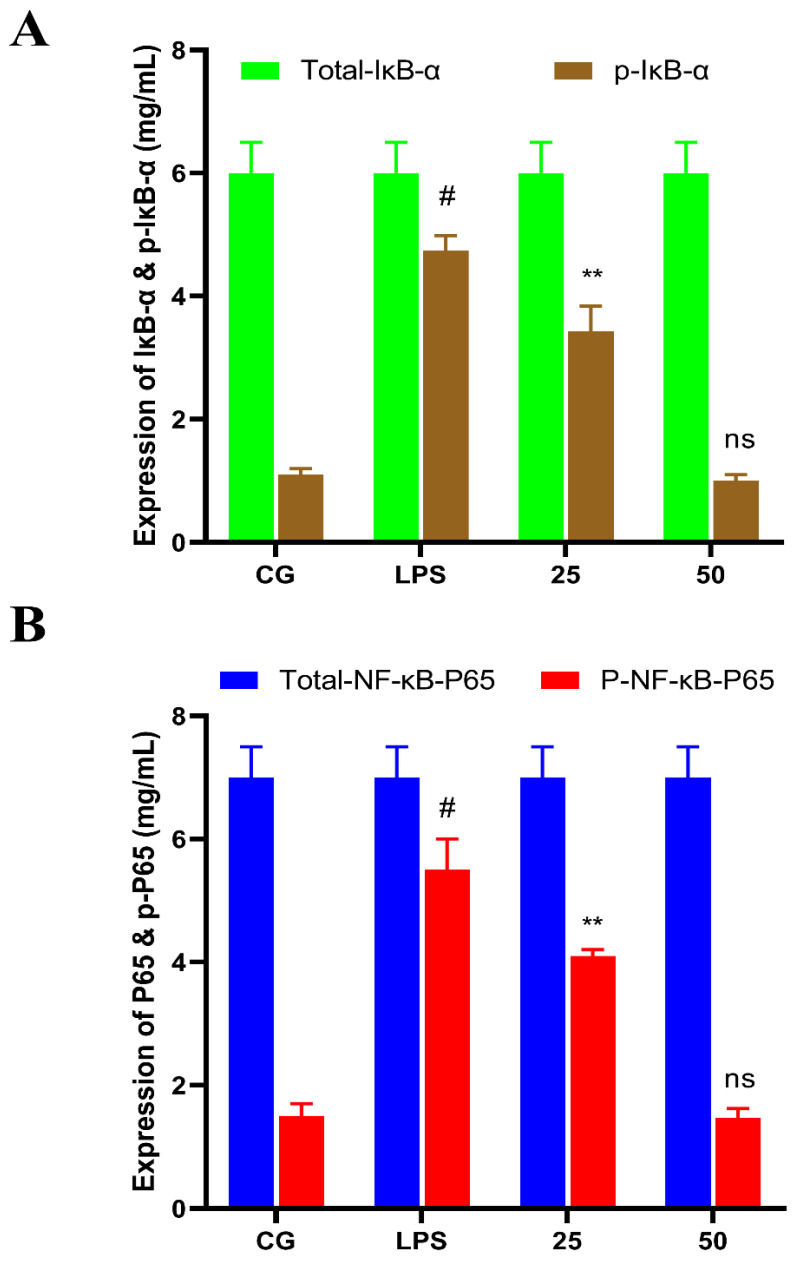
Effects of GnRb1 against LPS-induced expression NF-κB pathway proteins measured by ELISA. (**A**) The expression levels of the NF-κB p65 and its phosphorylated (p-NF-κB p65) form. (**B**) The expression levels of total IκBα and its phosphorylated (p-IκBα) form. CG represents the control group. LPS reveals the LPS-induced groups. The 25 and 50 are the GnRb1-administrated groups representing 25 mg/kg and 50 mg/kg per animal, respectively. GnRb1 indicates the Ginsenoside Rb1. Data statistics are demonstrated as mean ± S.E.M. The # *p* < 0.001 CG versus LPS group, ** *p* < 0.01, and ns indicates the nonsignificant difference between CG versus LPS + GnRb1 groups.

The anti-inflammatory properties of GnRb1 were investigated by determining the protein expression of NF-κB. The NF-κB pathway protein concentration was measured by ELISA assay. The protein expression of the phosphorylated form of IκB-α (Figure 7A) and NF-κB p65 (Figure 7B) protein were significantly higher (*p* < 0.05) in LPS-challenged groups. In contrast, the levels of these proteins were markedly decreased (*p* < 0.05) in GnRb1-treated groups.

## 4. Discussion

Pathogenesis triggered by microbes is important in the progression of inflammation [19]. Inflammation is a protective and defensive mechanism of the host immune system. However, massive inflammation might pose lethal effects on the structure and physiology of tissue. Endometritis is a major factor causing infertility in humans and animals [20]. Endometritis is characterized by foul-smelling and pyogenic uterine secretion associated with a high body temperature, depression, and dehydration [21]. LPS is derived from the cell wall of G-ve bacteria and is used widely to study mechanisms of inflammatory diseases [22,23,24]. In the recent era, bacterial resistance and drug residues owing to the undue usage of antibiotics have become a major concern globally for the dairy industry [25]. Therefore, there is currently a need to identify and develop new therapeutic agents that are more effective and safe to treat endometritis. Recently, traditional Chinese herbs have gained much attention due to their safe and heathy usage in food animals [24]. GnRb1 is a glycoside from the class of triterpenoid saponins, found in Panax ginseng, that has a long history of being used in China to cure various ailments. Its main biopharmacological properties includes cardioprotective [26,27], neuroprotective [28,29], nephroprotective [30], immunoregulatory [31], and anti-inflammatory properties [15,32,33]. GnRb1 was proved to suppress LPS-induced ALI [12]. Therefore, we presumed that GnRb1 might be a good anti-inflammatory herbal medicine. This is the first study to explore the anti-inflammatory properties of GnRb1 in a murine model of endometritis.

In the current study, hyperemia, hemorrhage, endothelial wall disruption, and massive neutrophil infiltration were observed in the LPS group. The LPS-stimulated histopathological changes were markedly alleviated by GnRb1 treatment. The characteristic pathological features of LPS-stimulated endometritis include infiltration of neutrophils, secretion of chemokines and cytokines, and edema [34]. MPO activity is a biomarker of the accumulation of neutrophils during acute inflammation caused by S. aureus [32,33] and E. coli [5,8]. The results indicated that MPO activity was increased in the LPS group. However, the elevated levels of MPO were suppressed by the administration of GnRb1, which means that GnRb1 suppress the inflammation in the uterine tissue. The uterine W/D ratio is sharply increased in LPS-challenged murine uteri, but W/D ratio is dramatically decreased upon GnRb1 therapy. It suggested that GnRb1 effectively alleviated endometritis by suppressing uterine edema and MPO activity.

TLR4 is a vital receptor to be activated in the LPS-triggered host defense mechanism. It is evidenced from the results of many studies that once TLR4 is activates, it triggers the activation of the downstream pathways, including the NF-κB pathway [35,36,37]. Therefore, it is hypothesized to determine whether the anti-inflammatory properties of GnRb1 will be achieved through the TLR4-mediated NF-κB pathway. It is revealed the LPS-challenged uteri experienced boosted gene and protein expression of TLR4, but that was suppressed by GnRb1 administration. The NF-κB pathway is one of the main crucial inflammatory pathways that is activated by G-ve bacteria via the regulation of TLR4 receptors [38,39]. During normal physiological conditions, NF-κB p65 and IκBα are localized in the cytoplasm of the cell [23]. Upon stimulation via TLRs, these proteins were phosphorylated and the NF-κB p65 subunit dissociated from IκBα. Phosphorylated NF-κB p65 is then transferred to the nucleus to regulate the production of many inflammatory mediators, including IL-1β, IL-6, and TNF-α [40,41]. The findings of the current study suggest that GnRb1 remarkably ameliorated the phosphorylation of the NF-κB p65 and IκBα.

Pro-inflammatory mediators perform an essential role in the development of inflammatory diseases, such as mastitis [34,42], ALI [43], and endometritis [5,44]. TNF-α is released from macrophages, monocyte, and T-lymphocytes during the initial stage of inflammation and has a role in neutrophil activation and the secretion of other pro-inflammatory cytokines [45]. IL-1β regulates the inflammatory responses, also produced from macrophages [46]. IL-6 is produced during stress, trauma, infections, etc., and is thought to maintain tissue injury [47]. The results of the present research indicated that pro-inflammatory mediators (IL-1β, IL-6, and TNF-α) in the uterine tissue were dose-dependently and dramatically repressed in the GnRb1 group. Surprisingly, it was observed that IL-10 was boosted in terms of expression during the LPS challenge, which shows that IL-10 is an anti-inflammatory cytokine. The elevated level of the IL-10 in GnRb1 groups dose-dependently suggested that GnRb1 has anti-inflammatory properties. These findings suggested that GnRb1-elicited anti-inflammatory effects might be due to the suppression of levels of pro-inflammatory cytokines. It was observed that GnRb1 protects from LPS-induced uterine inflammation. Therefore, it is believed that GnRb1 attenuates extensive inflammatory reactions triggered by LPS in uterine tissue via the suppression of the TLR4-mediated NF-κB pathway.

## 5. Conclusions

In conclusion, the current research is the first study to confirm that GnRb1 exerts protective effects against LPS-induced endometritis in mice. The protective properties of GnRb1 might be due to improvements in histopathological alterations, MPO activity, and uterine edema. Moreover, GnRb1 has the potential to inhibit the secretion of pro-inflammatory cytokines and enhance the production of anti-inflammatory mediators. Furthermore, the promising anti-inflammatory effect of GnRb1 was via the TLR4-mediated NF-κB pathway. Taken together, GnRb1 might be a potent anti-inflammatory drug to treat endometritis in both humans and animals.

## Figures and Tables

**Table 1:** The primers used for RT-qPCR assay.

Target Gene	Primer	Primer Sequence (5′→3′)	Accession No.	Product Size
*TLR4*	Forward	TTCAGAGCCGTTGGTGTATC	NM_021297.2	170
	Reverse	CTCCCATTCCAGGTAGGTGT		
*TNF-α*	Forward	CTTCTCATTCCTGCTTGTG	NM_013693.3	198
	Reverse	ACTTGGTGGTTTGCTACG		
*IL-1β*	Forward	CCTGGGCTGTCCTGATGAGAG	NM_008361.4	131
	Reverse	TCCACGGGAAAGACACAGGTA		
*IL-6*	Forward	GGCGGATCGGATGTTGTGAT	NM_031168.1	199
	Reverse	GGACCCCAGACAATCGGTTG		
*IL-10*	Forward	ACAGCCGGGAAGACAATAACT	NM_010548.2	66
	Reverse	GCAGCTCTAGGAGCATGTGG		
*GAPDH*	Forward	GTGGCAAAGTGGAGATTGTTG	NM_001289726.1	109
	Reverse	TTGACTGTGCCGTTGAATTTG		

## Data Availability

Data are contained within the article.

## Sample Availability

Samples of the compounds are not available from the authors.
